# Construction of an Evaluation System for Big Food Concept Education and Its Behavioral Impact Mechanism Among College Students—An Empirical Study Based on a Survey of Students

**DOI:** 10.3390/foods15040776

**Published:** 2026-02-21

**Authors:** Yong He, Ruirui Tang, Minlun Hu, Fang Chen, Xiaoqian Gao, Dandan Li, Yaowen Liu

**Affiliations:** College of Food Science, Sichuan Agricultural University, Ya’an 625014, China

**Keywords:** college students, big food concept, educational evaluation system, behavioral impact, mechanism of action

## Abstract

Education on the Big Food Concept, as a strategic framework for ensuring national food security and promoting high-quality agricultural development, represents a key nexus between ideological and political education and quality-oriented education for college students. Based on survey data from 1268 students across six provinces in China, this study utilized the Delphi method, the analytic hierarchy process (AHP), and structural equation modeling (SEM) to develop a four-dimensional evaluation system encompassing cognitive, affective, value, and behavioral dimensions. It examined the relationship and underlying mechanism through which Big Food Concept education influences student behavior. The results indicate that college students’ overall understanding of the Big Food Concept remains at a moderate level, with particularly limited awareness of diversified food supply systems. The weights of the dimensions in the educational evaluation system were as follows: behavioral dimension (0.342) > cognitive dimension (0.287) > value dimension (0.221) > affective dimension (0.150). Big Food Concept education shapes student behavior through the sequential pathway of cognitive enlightenment, affective resonance, and value internalization, with value internalization demonstrating the strongest mediating effect (β = 0.413, *p* < 0.001). The evaluation system developed in this study is a practical tool for assessing the effectiveness of Big Food Concept education in higher institutions, while the identified mechanism provides a theoretical basis for implementing targeted educational practices.

## 1. Introduction

“Among all matters, food is paramount.” Food security has always been the top priority in national governance [1,2]. The General Office of the State Council has promulgated the *Opinions on Practicing the Big Food Concept and Building a Diversified Food Supply System*, elevating the Big Food Concept to the level of national strategic importance [3,4]. The document explicitly calls for “guiding healthy and nutritious food consumption, and promoting sustained efforts in grain conservation and food waste reduction.” The Big Food Concept promotes a transition from the reliance on traditional grains to focus on the entire food supply from agriculture, forestry, animal husbandry, and fisheries [5]. It involves the synergy of multiple dimensions, including production, ecology, technology, and consumption. This represents a higher-level perspective on food security and embodies a sustainable development paradigm [6,7,8]. As the future backbone of societal development, college students’ understanding of food, consumption habits, and sense of responsibility are directly linked to the effective implementation of national food security strategies. These factors also significantly influence the broader agenda of building a Healthy China and advancing ecological civilization. As the primary base for talent cultivation, integrating the Big Food Concept into the ideological and political education and quality-oriented education systems of higher education institutions is both a necessary requirement for implementing national strategies and an innovative pathway for enhancing educational quality. However, in current practice, Big Food Concept education suffers from fragmentation and formalism, lacking a systematic educational content framework and scientific tools for evaluating effectiveness. Research on the internal mechanisms through which education influences student behavior is particularly underdeveloped. Existing surveys indicate that most college students are unfamiliar with the Big Food Concept, and many only have a vague understanding of its core principles, such as food source diversity and ecological sustainability. This is closely related to the insufficient provision of relevant education in universities. Therefore, constructing a scientific education evaluation system and clarifying how education influences student behavior have become urgent needs in promoting the standardization, effectiveness, and systematization of Big Food Concept education.

Research related to the Big Food Concept in China has been rapidly emerged since the introduction of the national strategy, and has formed three core research areas: conceptual interpretation and value exploration; practical exploration; and surveying the current situation. However, significant gaps remain in the areas of educational evaluation and mechanism research. Regarding research on connotation and value, scholars predominantly interpret the multidimensional principles of the Big Food Concept based on national policy orientation. They emphasize its core feature of transitioning from solely relying on traditional staple grains to seeking food from all territorial resources [9], clarify its integrated security principle encompassing “quantity, quality, nutrition, and ecology” [10], and highlight its multiple benefits in ensuring food security, promoting high-quality agricultural development, and advancing the Healthy China initiative [11,12]. Some studies, within the legislative context of the Food Security Guarantee Law, discuss the legal status and practical boundaries of the Big Food Concept, providing theoretical support for the legitimacy and centrality of its educational content [13]. However, most existing research has focused on macro-level policies and industrial aspects, and lacks targeted interpretation of the key cognitive and value aspects required for educating college students.

In the exploration of educational practice pathways, higher education institutions have tested a diversity of educational methods, forming a dual-track model of “curriculum-based ideological and political education + practical experience.” At the curriculum level, the Big Food Concept is integrated into specialized courses such as Food Chemistry and Oil and Fat Extraction Technology. At the practical level, institutions such as South China Agricultural University, Zhejiang University, Wuhan Polytechnic University, and China Agricultural University have implemented behavioral guidance strategies—including immersive experiences, hands-on labor practices [14,15,16], information-based interventions, commitment mechanisms, and smart campus canteens—to enhance education on the Big Food Concept [17]. In addition, the Chinese University of Hong Kong has included vegetarian/food education in its general education courses, including a course titled “Future of Food: Plant-Based Living”. Within the framework of the comprehensive food education system, Shanghai Ocean University has established an education system that includes content on the food industry with the aim of cultivating interdisciplinary and multi-skilled talents who not only possess knowledge in food science and engineering, but also in food economic management. Jiangnan University has built a high-level communication platform, innovated collaborative education mechanisms, and promoted the sharing of high-quality resources. Its aim is to construct a new educational paradigm with “ideological and political education as the core and integration of industry, education, and scientific and technological innovation as the “breakthrough”, promoting high-quality innovation in Chinese food disciplines and the construction of an education powerhouse in China. However, these practices are mostly scattered pilot projects, lacking a unified educational objective framework and effectiveness feedback mechanism, and there is no systematic educational system.

The existing studies employing current situation surveys and behavioral analysis primarily focused on two dimensions. First is college students’ awareness of the Big Food Concept, along with their nutritional knowledge and the amount of food waste that they generate [18]. A questionnaire survey of 9192 university students in China revealed that 74% generate food waste when they eat at campus canteens, with an average of 61.03 g wasted per meal per person. The top three food waste categories were wheat-based products (25.78%), rice (20.36%), and vegetables (18.61%) [19]. The second dimension is behavioral influencing factors. The research by Zhang Y. [20] based on the Theory of Planned Behavior indicated that perceived behavioral control and dietary behavioral intention are key variables influencing healthy food choices among university students. However, these studies did not treat Big Food Concept education as a core explanatory variable, nor did they address the influence of deep-seated value dimensions such as ecological responsibility and national strategy.

Research on evaluation systems and institutional mechanisms remains relatively underdeveloped, with most existing assessment tools limited to measuring a single dimension. For example, dietary health literacy questionnaires only cover information acquisition and application abilities, and do not take into account emotional resonance and value identification. A limited number of comprehensive evaluation studies have attempted to construct multidimensional frameworks, but most remain at the theoretical design stage without validation of their reliability and validity through large-sample empirical studies. The research on behavioral mechanisms is particularly weak. The existing literature primarily describes correlations between cognition and behavior without considering the transmission pathway of “education–cognition–emotion–value–behavior.” Quantitative analysis of the mediating and moderating effects at each stage is also lacking. There is no direct conceptual equivalent to the Big Food Concept in international discourse. Related foreign research is primarily concentrated in three major domains: food literacy education, sustainable food systems, and healthy dietary behaviors [21,22,23]. These areas have developed mature assessment tools and intervention models, yet these countries’ national strategy and goals fundamentally differ from those of China. In the realm of food literacy assessment and education, numerous well-established evaluation systems have been developed abroad. For instance, a food literacy scale validated for the university student population by Portuguese scholars comprises 26 items that cover three dimensions (nutritional knowledge, food label comprehension, and healthy dietary practices) and has demonstrated excellent reliability and validity (Cronbach’s α > 0.9) [24]. The NUTRIDIET questionnaire developed in Italy employs a dual-module design to simultaneously assess nutritional knowledge and health perceptions, and can be adapted to evaluate dietary patterns [25]. These tools focus on evaluating individual health and environmental knowledge but their dimensional design does not include evaluations of macro-level aspects such as national food security or strategic responsibility, making them unsuitable for studying the policy implications of China’s Big Food Concept [13,26].

Regarding interventions for sustainable food systems, university campuses are key sites for pilot projects. The research has focused on optimizing dietary behaviors and reducing environmental impacts. A systematic review and meta-analysis by the Johns Hopkins University found that multimodal interventions (combining information dissemination, environmental modification, and skills training) are significantly more effective than single-strategy approaches, achieving a success rate of 67.7% [27]. A study at a U.S. university integrating the “Real Food Challenge (RFC)” with the Sustainability Indicator Management and Analysis Platform (SIMAP) assessed the carbon emission impact of campus food procurement and found that a fully vegetarian scenario could reduce greenhouse gas emissions by 38.0% [28]. While these studies validate the effectiveness of environmental design and comprehensive interventions, their goals primarily centered on public health and environmental sustainability, without considering strategic needs such as national food security. In investigating the mechanisms underlying healthy dietary behavior, foreign research often constructs explanatory models based on theories such as the Theory of Planned Behavior and Social Cognitive Theory, which have revealed influencing factors that act at multiple levels. At the individual level, the predictive roles of perceived behavioral control and dietary intention on healthy food choices have been empirically supported [29]. At the environmental level, factors such as cafeteria meal offerings and dining environment design show significant guiding effects on behavior [30]. At the psychological level, affective factors such as food appreciation and ecological empathy play mediating roles in the transition from cognition to behavior [31]. However, the existing studies predominantly focused on the direct effects of individual and environmental factors. There is a lack of systematic exploration into the mediation pathway of “cognition–affect–values,” and behavioral analysis is seldom conducted within the context of specific national strategic frameworks, which is needed to understand social value orientations. A literature review incorporating domestic and international perspectives found that while foreign research has strengths such as mature assessment tools, rich empirical data, and precise intervention strategies, it exhibits three major shortcomings in terms of contextual applicability to China: First, a difference in value orientation exists; these studies lack focus on macro-strategic concerns such as national food security, which constitutes a core principle in China’s Big Food Concept. Second, a difference in cultural context is evident, as their assessment indicators and intervention strategies are rooted in Western dietary cultures and educational systems; this may lead to incompatibility if directly applied in China. Third, insufficient targeting of specific groups remains an issue; although specialized assessment tools for university students have been developed, they have not been optimized based on the value formation patterns and behavioral characteristics of the youth demographic [24]. Overall, while both domestic and international research provide a theoretical foundation and methodological references for Big Food Concept education, significant gaps remain. Domestically, there is a lack of systematic evaluation systems integrating cognition, affect, values, and behavior, and the chain mechanism through which education influences behavior has not been clarified. Internationally, the existing assessment tools and intervention strategies are difficult to adapt to the strategic principles and cultural context of China and its Big Food Concept. This study aims to effectively address these research gaps by constructing a localized evaluation system and revealing the multidimensional mechanism of action of Big Food Concept education.

This study extends the research perspective of the Big Food Concept from its conventional focus on agricultural production and policy to the educational domain and college student groups. By constructing a multidimensional, quantifiable educational evaluation system, it facilitates the theoretical exploration of the educational dimension within the field of Big Food Concept research. Furthermore, by revealing the mechanism through which education influences student behavior, it refines the theoretical framework of “education–cognition–behavior” transformation and provides a novel perspective for interdisciplinary research integrating ideological and political education into ecological civilization education. The developed evaluation system can be directly applied to accurately assess the effectiveness of Big Food Concept education in higher education institutions to identify targeted strategies for improving educational quality. The identified mechanism provides a practical basis for designing integrated educational programs that include the dimensions of cognition, emotion, values, and behavior, thereby supporting the cultivation of a new generation of college students equipped with food security awareness, healthy consumption concepts, and a sense of responsibility toward ecological civilization.

This study had three core objectives: (1) to construct and validate a four-dimensional (cognitive, affective, value-based, and behavioral) framework for evaluating Big Food Concept education for university students, and assessing its reliability, validity, and applicability; (2) to elucidate the mechanisms through which Big Food Concept education influences student behaviors, with a specific focus on the mediating roles of cognition, affect, and values; and (3) to provide theoretical foundations and practical recommendations for optimizing Big Food Concept education in higher education institutions and supporting the implementation of national food security strategies.

## 2. Research Design and Methods

### 2.1. Research Hypotheses

Based on the Theory of Planned Behavior (TPB) and the Value–Belief–Norm Theory (VBN), and considering the core principles of Big Food Concept education, the following research hypotheses were proposed. H1: Big Food Concept education has a significant positive effect on students’ cognitive enlightenment. H2: Big Food Concept education has a significant positive effect on students’ affective resonance. H3: Big Food Concept education has a significant positive effect on students’ value internalization. H4: Big Food Concept education has a significant positive effect on students’ behaviors. H5: Cognitive, affective, and value factors play a mediating role between Big Food Concept education and student behavior.

The TPB posits that cognitive factors, such as behavioral attitudes and subjective norms, serve as foundational antecedents to behavioral intention. This provides the theoretical basis for the core “cognition → behavior” pathway proposed by this study. The VBN emphasizes the central mechanism of “values → beliefs → personal norms → behavior”; based on this theory, this study proposes that values have a pivotal mediating role. Integrating the TPB and VBN produces a “cognition → affect → values → behavior” pathway where cognition provides the knowledge base for behavior; affect transforms rational cognition into emotional identification; internalized values turn external requirements into intrinsic pursuits; and behavior is the ultimate manifestation of this entire psychological process. This aligns closely with the “knowledge → affect → volition → practice” psychological mechanism in educational psychology and forms the core theoretical framework for this study’s structural equation model, which uses the sequence of “Big Food Concept education → cognitive enlightenment → affective resonance → value internalization → behavioral changes.” The core tenets of both the TPB and VBN are deeply embedded into the constructed SEM model to align the empirical model with theory.

### 2.2. Definition of the Big Food Concept

Based on policy documents and existing research (both domestic and international), this study defines the Big Food Concept as a strategic concept for ensuring food security that involves transcending the reliance on traditional staple foods, leveraging synergistic development across multiple sectors (including farming, forestry, animal husbandry, fisheries, and grassland industries), and integrating dimensions of production, ecology, technology, and consumption to achieve a diversified food supply and sustainable development.

### 2.3. Survey Participants

A multi-stage, stratified sampling method was adopted to select college students from 15 universities in six provinces in the eastern (Shandong and Jiangsu), central (Hubei and Jiangxi), and western (Sichuan and Chongqing) regions of China. The sample covered multiple disciplines, including agronomy, engineering, science, liberal arts, and management. The survey was conducted from May to December 2025. A total of 1500 questionnaires were distributed, with 1268 valid responses collected, yielding an effective response rate of 84.53% (Table 1). The basic characteristics of the sample are as follows: the gender distribution was 47.3% male and 52.7% female; the grade distribution was 28.6% freshmen, 31.2% sophomores, 24.5% juniors, and 15.7% seniors; the place of origin distribution was 42.1% inland rural areas, 35.8% inland urban areas, and 22.1% coastal urban areas; the highest proportion for monthly household income was in the range of CNY 5000–8000 (38.6%). The research protocol was reviewed and approved by the Sichuan Agricultural University Academic Ethics Committee, Sichuan Agricultural University (approval No. H20250026). Informed consent was obtained from all participants prior to their participation in the study. The confidentiality and privacy of the participants were ensured throughout the research process.

### 2.4. Research Methods

Delphi Method: Twelve experts (8 professors and 4 associate professors) from the fields of food science, ideological and political education, educational assessment, and agricultural policy were consulted during the development of the dimensional framework and specific indicators for the evaluation system. Two rounds of consultation were performed to ensure the scientific validity and rationality of the indicators. See the Supplementary Materials for the expert consultation questionnaire.

Analytic Hierarchy Process (AHP): A hierarchical structure comprising an objective level (Big Food Concept Education Evaluation system), criterion level (4 dimensions), and indicator level (16 specific indicators) was constructed for the analysis. Judgment matrices were created based on expert ratings, and the weights for each dimension and indicator were calculated accordingly.

Structural Equation Modeling (SEM): Using AMOS 24.0 software, a structural equation model was developed that included an educational variable, mediating variables (cognition, affect, and values), and an outcome variable (behavior). The model was used to examine the path relationships and mediating effects between the variables. Model fit was assessed through comparison to widely accepted criteria: χ^2^/df between 1 and 3, RMSEA < 0.08, GFI ≥ 0.9, AGFI ≥ 0.9, and CFI ≥ 0.9. The bias-corrected bootstrap method (using 5000 resamples) was used to test the mediation effects, with a 95% confidence interval excluding zero indicating significance. Furthermore, demographic variables—gender, major, grade level, place of origin, and monthly household income—were included as control variables to mitigate their potential confounding effects on the core pathway relationships.

Questionnaire Survey: Drawing on existing research scales and the core tenets of the Big Food Concept, semi-structured interviews were conducted with 20 university students and 5 experts. This process ensured that the indicators for the cognitive, affective, value, and behavioral dimensions accurately and unambiguously reflect the core principles of the Big Food Concept. Based on this, the Questionnaire on University Students’ Cognition and Behavior Regarding the Big Food Concept (see Supplementary Materials) was designed. Following a pilot survey (*n* = 150) and subsequent revisions, the final formal questionnaire comprising 42 items was developed. All items were scored using a 5-point Likert scale (1 = strongly disagree, 5 = strongly agree).

### 2.5. Principles for Constructing the Evaluation System

Systematic Principle: The assessment considers the complete mechanism of action of Big Food Concept education—from cognitive enlightenment and emotional identification to value internalization and behavioral practice—to ensure that it is a comprehensive evaluation.

Scientific Principle: The selection of indicators was grounded in theoretical foundations and validated by expert input to ensure that they closely align with the core principles of the Big Food Concept. The determination of the indicator weights employed rigorous quantitative methods.

Operational Principle: The selection of indicators balanced scientific rigor and practical applicability. All items are presented in a standardized scale format to facilitate data collection and statistical analysis.

Guiding Principle: Beyond evaluating effectiveness, the assessment is intended to guide educational practices, with a particular emphasis on the orienting role of the behavioral dimension.

## 3. Results and Discussion

### 3.1. Construction of the College Students’ Big Food Concept Education Evaluation System

#### 3.1.1. Dimensions and Indicator Setting for the Evaluation System

Based on a comprehensive review of the literature, expert consultations, and field investigations, a four-dimensional assessment framework comprising 16 specific indicators was established (Table 2).

Cognitive Dimension: This dimension reflects students’ understanding of the core principles of the Big Food Concept. It includes four indicators: awareness of the Big Food Concept, knowledge of diversified food supply systems, comprehension of food security strategies, and understanding of ecological sustainability.

Affective Dimension: This dimension captures students’ emotional attitudes toward food, labor, and nature. It encompasses four indicators: appreciation for food, gratitude for labor, ecological empathy, and agreement with relevant policies.

Value Dimension: This dimension represents students’ judgment and commitment to food-related values. It consists of four indicators: values related to healthy eating, values emphasizing conservation and environmental protection, values centered on responsibility and accountability, and values supporting synergistic development.

Behavioral Dimension: This dimension comprises students’ actual practices in dietary consumption and participation in related activities. It includes four indicators: food conservation behaviors, healthy consumption habits, ecological protection actions, and engagement in practical initiatives.

#### 3.1.2. Determination of Indicator Weights

The Analytic Hierarchy Process (AHP) was employed to determine the weights of each dimension and indicator; the specific steps are outlined below.

Construction of the Hierarchical Structure: The objective level (A) was defined as the College Students’ Big Food Concept Education Evaluation system. The criterion level (B) consisted of four dimensions: cognitive (B1), affective (B2), value (B3), and behavioral (B4) dimensions. The indicator level (C) included 16 specific indicators (Table 3).

Development of Judgment Matrices: Twelve experts performed pairwise comparisons of the importance of the elements of each hierarchical level using the 1–9 scale method; the results were used to create judgment matrices.

Weight Calculation and Consistency Verification: The weights of each dimension and indicator were calculated using the eigenvalue method. The consistency ratio (CR) was verified to be <0.1, indicating that the results are reliable.

The final weights and ranking of the dimensions were as follows: behavioral dimension (B4 = 0.342) > cognitive dimension (B1 = 0.287) > value dimension (B3 = 0.221) > affective dimension (B2 = 0.150). The top eight indicators were food conservation behavior (C13 = 0.098), understanding of the diversified food supply system (C2 = 0.087), healthy consumption behavior (C14 = 0.082), ecological protection behavior (C15 = 0.081), practical participation behavior (C16 = 0.081), responsibility and duty values (C10 = 0.076), understanding of food security strategies (C3 = 0.071), and understanding of the Big Food Concept (C1 = 0.066). This weight distribution reflects the educational goal of integrating knowledge and action, highlighting the central role of behavioral practice in educational assessment while also emphasizing the importance of cognitive foundations and value guidance.

#### 3.1.3. Establishment of Evaluation Criteria

Drawing on commonly used standards in educational assessments and considering the distribution of the survey data, educational effectiveness was categorized into four levels: Excellent (scores > 85), Good (scores of 70–84), Moderate (scores of 55–69), and Poor (scores < 55) (Table 4). Individuals with an Excellent score demonstrate a systematic understanding of the Big Food Concept, exhibit stable emotional identification and value adherence, and consistently practice healthy, economical, and ecologically sound behaviors. Those with a Good score possess relatively comprehensive cognition, show positive emotional and value inclinations, and display fairly stable behavioral performance. Participants with a Moderate score have basic knowledge but lack depth, exhibit vague emotional and value identification, and their behaviors tend to be incidental or inconsistent. Individuals with a Poor score show deficient cognition, demonstrate negative emotional and value orientations, and engage in behaviors such as wasting food and unhealthy consumption.

### 3.2. Current Big Food Concept Education Effects on College Students and Validation of the Education Evaluation System

#### 3.2.1. Analysis of the Current Effects of Big Food Concept Education on College Students

The current effects of Big Food Concept education on college students were evaluated using the survey data and evaluation system (Table 5). The results show that the overall mean score of the sample was 63.72 points, falling within the moderate level. The mean scores for each dimension were as follows: 61.38 points for the cognitive dimension; 65.42 points for the affective dimension; 64.15 points for the value dimension; and 62.87 points for the behavioral dimension. The specific results are outlined below.

**Figure 1:**
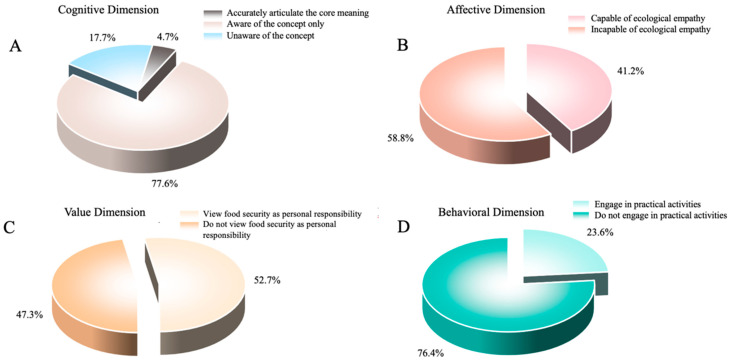
Current effects of Big Food Concept education on college students: (**A**) cognitive dimension; (**B**) affective dimension; (**C**) value dimension; (**D**) behavioral dimension.

Cognitive Dimension: Conceptual awareness is emerging but lacks depth (Figure 1A). While 82.3% of students reported having heard of the Big Food Concept, only 4.7% could accurately articulate its core meaning. Awareness of staple food security was relatively high (78.6% correctly answered questions related to food self-sufficiency rates). However, less than 30% demonstrated an understanding of diversified supply systems such as the “Forest Food Bank” and “Blue Granary,” and comprehension of the relationship between food production and ecological protection remained vague. This cognitive gap may constrain students’ understanding of the extended dimensions of food security strategies, hinder their acceptance of non-traditional food sources (e.g., forest foods and aquatic products), and ultimately impede the implementation of the national strategy to “build a diversified food supply system.” The fundamental cause of this gap lies in the insufficient integration of content related to non-staple food supplies within the current Big Food Concept curriculum in higher education institutions.

Affective Dimension: Positive emotions predominate, but resonance is limited (Figure 1B). A majority of students expressed cherishing food (76.8%) and gratitude toward farmers’ labor (69.3%). Nevertheless, only 41.2% exhibited ecological empathy toward food resource conservation, and emotional acceptance of national food security policies showed regional variations, with higher levels of acceptance among students from coastal areas compared to those from inland regions.

Value Dimension: Health and frugality values are prominent, while a sense of responsibility has not fully developed (Figure 1C). Most students endorsed values related to healthy eating (86.5%) and food conservation (79.8%). However, only 52.7% regarded food security as a personal responsibility, and merely 43.5% expressed concern about the sustainability of food production.

Behavioral Dimension: Basic behaviors are relatively satisfactory, but practical engagement is insufficient (Figure 1D). Daily food conservation was reported by 68.4% of students, and 72.1% indicated a tendency to choose healthy foods. However, only 23.6% participated in practical activities such as campus farm work or food waste surveys, and fewer than 15% actively promoted the Big Food Concept.

#### 3.2.2. Reliability and Validity Testing of the Evaluation System

Reliability Test: The overall scale showed a Cronbach’s α coefficient of 0.892. The α coefficients were 0.843, 0.817, 0.832, and 0.851 for the cognitive, affective, value, and behavioral dimensions, respectively. All values exceeded the threshold of 0.8, indicating good internal consistency of the scale. The composite reliability (CR) values were all above 0.8, and the average variance extracted (AVE) values were all above 0.5, further confirming the reliability of the evaluation system (Table 6).

Validity Test: Content validity was verified through the expert consultation method. Twelve experts evaluated the representativeness and relevance of the indicators and a consensus rate of 86% was achieved. Structural validity was examined using confirmatory factor analysis (CFA). The model fit indices met the ideal standards: χ^2^/df = 2.37, RMSEA = 0.038, GFI = 0.926, AGFI = 0.904, and CFI = 0.948. These results indicate a good fit between the scale structure and the theoretical model, supporting the structural validity of the evaluation system (Table 6).

#### 3.2.3. Applicability Analysis of the Evaluation System

The survey sample was grouped by university major (comprehensive, agriculture and forestry, science and engineering, humanities, and social sciences), grade level, and place of origin. Analysis of variance (ANOVA) was performed to examine the applicability of the evaluation system across these groups. The results revealed significant differences in scores across the dimensions of the evaluation system among different groups (*p* < 0.05). Students from agriculture and forestry universities scored significantly higher on the cognitive dimension compared to students from other majors (F = 4.32, *p* < 0.01). Upper-year students obtained significantly higher scores in the behavioral dimension than lower-year students (F = 3.76, *p* < 0.01). Furthermore, students from rural backgrounds scored significantly higher on the affective dimension than their urban counterparts (F = 3.21, *p* < 0.05). These findings indicate that the evaluation system can effectively differentiate among these distinct groups, confirming good applicability.

### 3.3. Impact of Big Food Concept Education on Student Behavior and Its Mechanism of Action

#### 3.3.1. Definition of Research Variables

Independent Variable: Big Food Concept education (X) was measured using four items assessing the frequency and depth of students’ exposure to such education, covering course instruction, practical activities, awareness campaigns, and cultural immersion (Table 7).

Mediating Variables: The mediating variables were the cognitive (M1), affective (M2), and value (M3) dimensions, each measured using four items reflecting students’ understanding of, emotional attitudes toward, and values related to the Big Food Concept.

Dependent Variable: The behavioral dimension (Y) was measured using four items assessing healthy consumption behavior, food conservation behavior, ecological protection behavior, and practical participation behavior, which collectively reflect students’ actual behavioral performance.

#### 3.3.2. Model Construction and Fit

A chain mediation model was constructed, hypothesizing that Big Food Concept Education influences student behavior through three pathways: “X → M1 → M3 → Y,” “X → M2 → M3 → Y,” and “X → M1 → M2 → M3 → Y.” The model was estimated using AMOS 24.0. The results show that the model fit indices were as follows: χ^2^/df = 2.13, RMSEA = 0.035, GFI = 0.931, AGFI = 0.908, CFI = 0.952. These values all fall within the acceptable ranges, indicating an excellent fit of the model to the observed data.

#### 3.3.3. Analysis of Impact Relationships

Analysis of Direct Effect: Big Food Concept education exerted a significant, direct, positive effect on student behavior (β = 0.237, *p* < 0.001), indicating that strengthening this education can directly promote the practice of healthy, economical, and ecological behaviors. Education also had significant positive effects on the cognitive dimension (β = 0.528, *p* < 0.001), affective dimension (β = 0.364, *p* < 0.001), and value dimension (β = 0.289, *p* < 0.001), validating hypotheses H1–4.

Analysis of Indirect Effects: The bias-corrected bootstrap method (using 5000 resamples) was used to test the indirect effects. The results showed that the indirect effects via the cognitive, affective, and value dimensions were significant (total indirect effect = 0.586, 95% CI [0.521, 0.653], excluding zero). The specific indirect effects for the three paths were as follows: X → M1 → M3 → Y (effect = 0.207, 95% CI [0.162, 0.253]), X → M2 → M3 → Y (effect = 0.174, 95% CI [0.131, 0.218]), and X → M1 → M2 → M3 → Y (effect = 0.205, 95% CI [0.158, 0.252]). The indirect effect through the value dimension (i.e., M3) was the most substantial (β = 0.413, *p* < 0.001), indicating that value internalization is a key mechanism linking education to behavior.

### 3.4. Explanation of the Action Mechanism

Based on the empirical findings and the essential characteristics of Big Food Concept education, its influence on student behavior can be summarized as the following mechanism: “cognitive enlightenment → affective resonance → value internalization → behavioral practice.”

Cognitive Enlightenment: Through classroom instruction and knowledge dissemination, Big Food Concept education imparts knowledge regarding its core principles, national food security strategies, and diversified food supply systems. This addresses students’ cognitive gaps and helps construct a systematic framework for understanding food. As the foundational step in the influence of education on behavior, it provides the cognitive basis for subsequent emotional identification and value formation.

Emotional Resonance: Through practical experiences (e.g., campus labor and visits to food supply chains) and emotional narratives (e.g., stories of labor behind food and ecological conservation cases), students’ feelings of cherishing food, gratitude towards laborers, and empathy for the ecological environment are evoked. This transforms cognitive understanding into emotional identification, thereby laying an emotional groundwork for value internalization.

Value Internalization: Under the combined influence of cognition enlightenment and emotional resonance, students gradually develop values concerning healthy eating, resource conservation, responsibility, and ecological synergy. This process converts the external requirements of the Big Food Concept into an internal pursuit of values, acting as the critical bridge that links cognition and emotion with behavioral practice. It thereby determines the stability and sustainability of the evoked behaviors.

Behavioral Practice: Internalized values guide students in making rational choices in their daily dietary consumption and practical participation. This manifests as behaviors such as healthy consumption, food conservation, participation in ecological protection, and promotion of the Big Food Concept, thereby achieving the educational goal of “integrating knowledge with action”.

## 4. Conclusions

Drawing on an empirical survey of over a thousand students, this study developed an evaluation framework and identified a behavioral impact mechanism. The results clearly indicate that Chinese university students’ overall understanding of the Big Food Concept is at a moderate level. The developed evaluation framework can be used by higher education institutions to identify targeted areas for educational improvement. Moreover, the research findings can help promote education as a key tool for cultivating public health literacy, enhancing students’ awareness of food conservation and waste reduction, solidifying the societal cognitive foundation for food security, and fostering a social ethos conducive to green development.

This study developed and validated a system for evaluating the impact of Big Food Concept education on college students, which encompassed cognitive, affective, value, and behavioral dimensions. The system demonstrated good reliability, validity, and applicability, and can thus be utilized as a scientific tool for evaluating educational effectiveness. Notably, the weight ranking of the dimensions (behavioral > cognitive > value > affective) reflects an educational orientation that prioritizes the integration of knowledge and action. The findings indicate that college students’ overall grasp of the Big Food Concept remains at a moderate level and is characterized by specific shortcomings: superficial cognition regarding diversified supply systems, a lack of deep ecological empathy, weak awareness of responsibility, and low levels of participation in practical behaviors. This profile can be summarized as “superficial cognition, weak affective connection, inadequate value internalization, and insufficient behavioral practice.” Furthermore, the impact of Big Food Concept education on student behavior was found to follow the mechanism of cognitive enlightenment → affective resonance → value internalization → behavioral practice. Among these stages, value internalization demonstrated the most significant mediating effect, serving as the key bridge in the process. In this framework, cognitive enlightenment acts as the foundation, affective resonance as the connecting link, and behavioral practice is the ultimate goal. This result indicates that although Big Food Concept enjoys a relatively high popularity among the public, the proportion with an accurate understanding of its meaning is relatively low. This phenomenon may reflect a certain degree of ambiguity and misunderstanding in the public’s perception of the food industry. In addition, the current low-diversity food system is the main target of the Big Food Concept as it promotes reliance on a single food source; nutritional imbalances; a low resource utilization efficiency; and backward production methods.

To refine the educational content system and strengthen cognitive enlightenment, the following steps are proposed: First, the Big Food Concept should be integrated into core university curricula, including ideological and political theory and general education courses, with dedicated modules such as “The Big Food Concept and National Security.” Second, the identified cognitive gaps should be targeted by emphasizing instruction on diversified food supply systems and the food production–ecology nexus, employing case studies and multimedia resources to enhance the depth of coverage. Third, accessible popular science materials should be developed and campus new media should be leveraged for ongoing knowledge dissemination. Innovating educational practice formats is crucial to enhancing the authenticity of affective resonance. This can be achieved by establishing a collaborative campus–society–family system that arranges visits to food supply chains and other practical activities; developing on-campus experiential learning sites (e.g., farms and waste recycling bases) for hands-on labor; and organizing engaging activities such as “Food Story Sharing Sessions” to foster emotional connection. To strengthen value guidance and promote deeper value internalization, a multi-pronged approach is recommended: aligning Big Food Concept education with broader socialist core values, emphasizing responsibility and ecological civilization; employing pedagogical strategies like role-modeling and scenario simulation to navigate value conflicts; and instituting long-term incentive mechanisms to recognize exemplary student practice, thereby cultivating a campus culture that values health, conservation, and ecology. Finally, improving the educational assessment mechanism will enhance the impact of practical activities. This could involve the following: formally incorporating the developed evaluation system into university quality evaluation frameworks for regular monitoring; utilizing assessment data to identify and address weaknesses in content and delivery; and establishing a dynamic adjustment process to update indicators and plans in response to evolving policy and student cognition.

Although this study has developed a systematic evaluation framework and revealed the underlying mechanisms, several limitations should be acknowledged. First, while the sample covered six provinces, its regional distribution requires further balancing, and the overall sample coverage needs to be expanded. Second, the proposed “education → cognition → affect → values → behavior” mechanism represents a theoretically supported mechanism based on logic and existing theories rather than conclusive proof of causality, thus preventing definitive causal claims. Third, the expert-assigned weights may have been influenced by academic and policy orientations, and the student scores could have been subject to self-reporting bias. Fourth, the cross-sectional design limits the ability to capture long-term dynamics of the educational impact. Finally, the mechanism analysis did not account for the moderating effects of individual differences, such as differences in major, gender, and upbringing. Future research could address these limitations by expanding the sample size and improving regional representativeness, adopting longitudinal or panel designs, integrating both expert- and student-oriented weighting approaches, and conducting in-depth analyses of the moderating role of individual differences, thereby refining both the evaluation framework and the theoretical mechanism.

## Figures and Tables

**Table 1:** Basic characteristics of participants.

Classification Item	Classification Category	Frequency (n)	Percentage (%)
Gender	Male	599	47.3
	Female	669	52.7
Grade	Freshman	363	28.6
	Sophomore	396	31.2
	Junior	311	24.5
	Senior	198	15.7
Place of Origin	Inland Rural	534	42.1
	Inland Urban	454	35.8
	Coastal Urban	280	22.1
Monthly Household Income	CNY ≤ 5000	276	21.8
	CNY 5001–8000	489	38.6
	CNY 8001–12,000	325	25.6
	CNY ≥ 12,001	178	14.0
Exposure to Big Food Concept Education	Never	398	31.4
	Occasionally	625	49.3
	Frequently	245	19.3

Note: This study obtained approval from the Ethics Review Committee of Sichuan Agricultural University (Approval No. H20250026). All participants voluntarily participated in the survey, and the collected data were kept strictly confidential and used solely for academic analysis.

**Table 2:** Assessment system for effects of Big Food Concept education on college students.

Dimension Name (A)	Dimension Description (B)	Specific Indicators (C)
Cognitive Dimension	Reflects students’ understanding of the core principles of the Big Food Concept	1. Understanding of the Big Food Concept 2. Understanding of Diversified Food Supply Systems 3. Understanding of Food Security Strategies 4. Understanding of Ecological Sustainability
Affective Dimension	Captures students’ emotional attitudes toward food, labor, and nature	5. Food Appreciation 6. Labor Gratitude 7. Ecological Empathy 8. Policy Agreement
Value Dimension	Represents students’ judgment and adherence to food-related values	9. Healthy Eating Values 10. Responsibility and Duty Values 11. Conservation and Environmental Protection Values 12. Synergistic Development Values
Behavioral Dimension	Comprises students’ actual performance in dietary consumption and practical participation in Big Food Concept-related activities	13. Food Conservation Behavior 14. Healthy Consumption Behavior 15. Ecological Protection Behavior 16. Practical Participation Behavior

**Table 3:** Weight distribution for indicators.

Indicator Code	Specific Indicator	Dimension	Indicator Weight	Weight Ranking
C13	Food Conservation Behavior	Behavioral Dimension	0.098	1
C2	Understanding of Diversified Food Supply Systems	Cognitive Dimension	0.087	2
C14	Healthy Consumption Behavior	Behavioral Dimension	0.082	3
C15	Ecological Protection Behavior	Behavioral Dimension	0.081	4
C16	Practical Participation Behavior	Behavioral Dimension	0.081	5
C10	Responsibility and Duty Values	Value Dimension	0.076	6
C3	Understanding of Food Security Strategies	Cognitive Dimension	0.071	7
C1	Understanding of the Big Food Concept	Cognitive Dimension	0.066	8
C4	Understanding of Ecological Sustainability	Cognitive Dimension	0.063	9
C9	Healthy Eating Values	Value Dimension	0.050	10
C11	Conservation and Environmental Protection Values	Value Dimension	0.048	11
C12	Synergistic Development Values	Value Dimension	0.047	12
C5	Food Appreciation	Affective Dimension	0.040	13
C6	Labor Gratitude	Affective Dimension	0.039	14
C7	Ecological Empathy	Affective Dimension	0.036	15
C8	Policy Agreement	Affective Dimension	0.035	16

**Table 4:** Statistics for dimension scores (n = 1268).

Dimension	Number of Items	Total Score Range	Mean Score (M)	Standard Deviation (SD)	Dimension Score (%)	Dimension Weight	Weight Ranking
Cognitive	12	12–60	3.07	0.68	61.38	0.287	2
Affective	10	10–50	3.27	0.59	65.42	0.150	4
Value	10	10–50	3.21	0.62	64.15	0.221	3
Behavioral	10	10–50	3.14	0.71	62.87	0.342	1
Overall Score	42	42–210	3.19	0.56	63.72	1.000	—

Note: 1. Dimension Score Rate = (Mean Score/Full Score per Item of the Dimension) × 100%, with each item rated on a 5-point scale. Dimension weights were calculated using the Analytic Hierarchy Process (AHP); all consistency ratios (CR) were < 0.1, meeting the consistency requirement. 2. Overall Score = Σ(Dimension Score × Corresponding Dimension Weight). The evaluation levels are defined as follows: Excellent (score > 85), Good (70 ≤ score ≤ 84), Moderate (55 ≤ score ≤ 69), and Poor (score < 55). 3. Each item was measured using a 5-point Likert scale. The “total dimension score range” was calculated as “number of items × 1 (minimum) to number of items × 5 (maximum)” (e.g., Cognitive Dimension: 12 items × 1 = 12; 12 items × 5 = 60, which is consistent with the table). The “mean (M)” is the average score per item (e.g., Cognitive Dimension M = 3.07, i.e., the average score across the 12 items). The “dimension score rate” was calculated as (item mean/5) × 100% (e.g., 3.07/5 × 100% = 61.38%).

**Table 5:** Statistics on specific manifestations of Big Food Concept education effects on College Students (n = 1268).

Dimension	Specific Manifestation	Awareness/Agreement Rate (%)
Cognitive	Have heard of the Big Food Concept	82.3
	Can accurately articulate its core meaning	4.7
	Understand staple food security-related issues	78.6
	Understand diversified supply systems (e.g., “Forest Food Bank,” “Blue Granary”) *	<30.0
Affective	Cherish food	76.8
	Express gratitude for farmers’ labor	69.3
	Experience ecological empathy for food resource protection	41.2
	Agree with national food security policies	Significant regional variation (coastal > inland)
Value	Identify with healthy eating values	86.5
	Emphasize food conservation	79.8
	View food security as a personal responsibility	52.7
	Concerned about the sustainability of food production	43.5
Behavioral	Practice daily food conservation	68.4
	Tend to choose healthy foods	72.1
	Participate in Big Food Concept-related practical activities	23.6
	Actively promote the concept of the Big Food Concept *	<15.0

Note: Data reflect responses scoring ≥3 points; items with an asterisk (*) have scores ≤2 points.

**Table 6:** Results of scale reliability and validity tests (n = 1268).

Dimension	Cronbach’s α Coefficient	Composite Reliability (CR)	Average Variance Extracted (AVE)	Expert Consensus Coefficient
Cognitive	0.843	0.865	0.532	0.86
Affective	0.817	0.841	0.518	0.86
Value	0.832	0.857	0.526	0.86
Behavioral	0.851	0.872	0.541	0.86
Overall Scale	0.892	0.905	0.524	0.86

**Table 7:** Results of indirect effect analysis (n = 1268, bootstrap resamples = 5000).

Influence Path	Effect Value	β Value	95% Confidence Interval (CI)	Significance (p)
Direct Effect (X → Y)	0.237	0.237	[0.185, 0.289]	<0.001
Total Indirect Effect	0.586	—	[0.521, 0.653]	<0.001
Path 1 (X → M1 → M3 → Y)	0.207	0.324	[0.162, 0.253]	<0.001
Path 2 (X → M2 → M3 → Y)	0.174	0.286	[0.131, 0.218]	<0.001
Path 3 (X → M1 → M2 → M3 → Y)	0.205	0.318	[0.158, 0.252]	<0.001
Indirect Effect of Value Dimension (M3 → Y)	—	0.413	[0.352, 0.474]	<0.001

Note: X = Big Food Concept education (independent variable), M1 = cognitive dimension, M2 = affective dimension, M3 = value dimension, Y = behavioral dimension. β values represent standardized path coefficients. Effect values were calculated using the bias-corrected bootstrap method.

## Data Availability

The original contributions presented in the study are included in the article/Supplementary Materials, further inquiries can be directed to the corresponding authors.

## Supplementary Materials

The following supporting information can be downloaded at: https://www.mdpi.com/article/10.3390/foods15040776/s1.
