# Supplementary material for: Construction of an Evaluation System for Big Food Concept Education and Its Behavioral Impact Mechanism Among College Students—An Empirical Study Based on a Survey of Students
Source: Foods. 2026 Feb 21;15(4):776. doi: 10.3390/foods15040776 (PMC12941065; doi:10.3390/foods15040776)
Supplement: Supplementary file 1 [file foods-15-00776-s001.zip › foods-4111499-supplementary.pdf]

Expert Consultation Questionnaire on the Construction of a College Students' Big Food Concept Education Evaluation System

(First Round)

Dear Experts,

Greetings! This research aims to construct a scientific and feasible evaluation system for college students' Big Food Concept education, providing tool support for assessing the effectiveness of such education in universities. Based on literature review and preliminary research, the preliminary evaluation system consists of 4 dimensions and 16 specific indicators. We invite you to compare the importance of the indicators pairwise using the Delphi method and put forward optimization suggestions based on your professional experience. Your professional judgment is the core guarantee of the research's scientificity. We sincerely request you to fill in this questionnaire carefully and thank you for your strong support!

I. Filling Instructions

1. Hierarchy of the Evaluation System: The target layer is "College Students' Big Food Concept Education Evaluation System"; the criterion layer includes four core dimensions: Cognitive Dimension, Affective Dimension, Value Dimension, and Behavioral Dimension; the indicator layer consists of 16 specific indicators (see details in the Indicator Explanation).
2. Importance Scale Rules (1-9 Scale Method):

Scale Value	Meaning	Reverse Scale Example
1	Two elements are equally important	If A is 1 to B, then B is 1 to A
3	The former is slightly more important than the latter	If A is 3 to B, then B is 1/3 to A
5	The former is obviously more important than the latter	If A is 5 to B, then B is 1/5 to A
7	The former is strongly more important than the latter	If A is 7 to B, then B is 1/7 to A
9	The former is extremely more important than the latter	If A is 9 to B, then B is 1/9 to A

| 2, 4, 6, 8 | Intermediate values between the adjacent scales above | Corresponding reverse intermediate values (e.g., if A is 2 relative to B, then B is 1/2 relative to A) |

II. Basic Information of Experts

1. Research Field: □ Food Science □ Ideological and Political Education □ Education Evaluation □ Agricultural Policy □ Others ________
2. Professional Title: □ Professor □ Associate Professor □ Others ________

3. Work Experience: □ Less than 10 years □ 10-20 years □ More than 20 years
4. Familiarity with the Field of Big Food Concept: □ Very Familiar □ Relatively Familiar □ General □ Unfamiliar

III. Indicator Explanation

**Criterion Layer (Dimensions)**	**Indicator Layer (Specific Indicators)**	**Indicator Connotation**
Cognitive Dimension (B1)	Cognition of the Big Food Concept (C1)	Degree of understanding of the core definition and core characteristics of the Big Food Concept
	Cognition of the Diversified Food Supply System (C2)	Cognition of "full-field supply of agriculture, forestry, animal husbandry, and fishery", "forest grain depot", "blue granary", etc.
	Cognition of the Food Security Strategy (C3)	Understanding of national food security policies, self-sufficiency rate goals, etc.
	Cognition of Ecological Sustainability (C4)	Cognition of the coordinated relationship between food production and ecological protection
Affective Dimension (B2)	Emotion of Food Cherishing (C5)	Attitude of valuing food resources and emotional tendency to oppose waste
	Emotion of Gratitude for Labor (C6)	Respect and gratitude towards food producers
	Emotional Empathy for Ecology (C7)	Empathy and concern for food resource protection and ecological balance
	Emotional Identification with Policies (C8)	Emotional recognition and support for national policies related to the Big Food Concept
Value Dimension (B3)	Healthy Diet Values (C9)	Tendency to take balanced nutrition and healthy consumption as the core values of diet
	Sense of Responsibility Values (C10)	Value judgment that regards ensuring food security as one's own social responsibility
	Values of Food Conservation and Environmental Protection (C11)	Value orientation advocating food conservation and reduction of resource consumption

Criterion Layer (Dimensions)	Indicator Layer (Specific Indicators)	Indicator Connotation
	Values of Coordinated Development (C12)	Recognition of the value concept of "production-consumption-ecology" coordinated development
	Food Conservation Behavior (C13)	Actual performance of reducing waste and ordering reasonably in daily diet
Behavioral Dimension (B4)	Healthy Consumption Behavior (C14)	Consumption behavior of choosing nutritionally balanced, green and safe food
	Ecological Protection Behavior (C15)	Practical behaviors such as participating in garbage classification and supporting environmentally friendly food production
	Practical Participation Behavior (C16)	Behaviors of participating in research, labor practice, and publicity related to the Big Food Concept

IV. Pairwise Comparison Judgment Matrix for Importance (Please Fill in Scale Values)

(I) Pairwise Comparison of the Criterion Layer (4 Dimensions)

Criterion Layer Elements	Cognitive Dimension (B1)	Affective Dimension (B2)	Value Dimension (B3)	Behavioral Dimension (B4)
Cognitive Dimension (B1)	-			
Affective Dimension (B2)	-	-		
Value Dimension (B3)	-	-	-	
Behavioral Dimension (B4)	-	-	-	-

(II) Pairwise Comparison of the Indicator Layer (Classified by Criterion Layer)

1. Comparison of Indicators under the Cognitive Dimension (B1)

Cognitive Dimension Indicators	Cognition of the Big Food Concept (C1)	Cognition of the Diversified Food Supply System (C2)	Cognition of the Food Security Strategy (C3)	Cognition of Ecological Sustainability (C4)
Cognition of the Big Food Concept (C1)	-			
Cognition of the Diversified Food Supply System (C2)	-	-		
Cognition of the Food Security Strategy (C3)	-	-	-	

Cognitive Dimension Indicators	Cognition of the Big Food Concept (C1)	Cognition of the Diversified Food Supply System (C2)	Cognition of the Food Security Strategy (C3)	Cognition of Ecological Sustainability (C4)
Cognition of Ecological Sustainability (C4)	-	-	-	-

2. Comparison of Indicators under the Affective Dimension (B2)

Affective Dimension Indicators	Emotion of Food Cherishing (C5)	Emotion of Gratitude for Labor (C6)	Emotional Empathy for Ecology (C7)	Emotional Identification with Policies (C8)
Emotion of Food Cherishing (C5)	-			
Emotion of Gratitude for Labor (C6)	-	-		
Emotional Empathy for Ecology (C7)	-	-	-	
Emotional Identification with Policies (C8)	-	-	-	-

3. Comparison of Indicators under the Value Dimension (B3)

Value Dimension Indicators	Healthy Diet Values (C9)	Sense of Responsibility Values (C10)	Values of Food Conservation and Environmental Protection (C11)	Values of Coordinated Development (C12)
Healthy Diet Values (C9)	-			
Sense of Responsibility Values (C10)	-	-		
Values of Food Conservation and Environmental Protection (C11)	-	-	-	
Values of Coordinated Development (C12)	-	-	-	-

4. Comparison of Indicators under the Behavioral Dimension (B4)

Behavioral Dimension Indicators	Food Conservation Behavior (C13)	Healthy Consumption Behavior (C14)	Ecological Protection Behavior (C15)	Practical Participation Behavior (C16)
Food Conservation Behavior (C13)	-			
Healthy Consumption Behavior (C14)	-	-		
Ecological Protection Behavior (C15)	-	-	-	
Practical Participation Behavior (C16)	-	-	-	-

V. Feedback and Supplementary Suggestions

1. Do you think the 4 dimensions of the evaluation system are complete? Is there a need to add, merge, or delete dimensions? Please explain:

2. Do you think the 16 specific indicators can fully reflect the connotation of the corresponding dimensions? Is there a need to add, delete, or modify indicators? Please explain:

3. Do you have any other optimization suggestions for the design of this indicator importance comparison?

4. Other supplementary comments:

Thank you again for your valuable time and professional support!

Research Group of "Construction of College Students' Big Food Concept Education Evaluation System and Research on Behavioral Impact Mechanism"

Expert Consultation Questionnaire on the Construction of a College Students' Big Food Concept Education Evaluation System

(Second Round)

Dear Experts,

Greetings! Thank you for participating in the first round of expert consultation! Based on the feedback from 12 experts in the first round, we have sorted out and optimized the indicators of the evaluation system (no adjustments to the indicators, and the expert consistency coefficient reached 86%). The core statistical results of the first round of consultation are fed back as follows. We kindly request you to combine these results to compare the importance of the indicators pairwise again, and further confirm or adjust your judgments to ensure the scientificity and rationality of the evaluation system.

I. Feedback on Core Results of the First Round of Consultation

1. Preliminary Weight Ranking of Dimensions: Affective Dimension (0.152) < Value Dimension (0.219) < Cognitive Dimension (0.291) < Behavioral Dimension (0.338) (Consistency Ratio CR = 0.072 < 0.1, indicating reliable results).
2. Top 8 Indicators by Preliminary Weight: Food Conservation Behavior (0.096), Cognition of the Diversified Food Supply System (0.085), Healthy Consumption Behavior (0.083), Ecological Protection Behavior (0.080), Practical Participation Behavior (0.079), Sense of Responsibility Values (0.075), Cognition of the Food Security Strategy (0.070), Cognition of the Big Food Concept (0.067).
3. Focus of Expert Opinions: 91.7% of experts believe the dimension setting is complete, and 83.3% of experts recognize the representativeness of the 16 indicators. It is suggested to focus on optimizing the discrimination degree of indicator importance.

II. Filling Instructions

1. The hierarchy of the evaluation system and the connotation of indicators are consistent with the first round (see Indicator Explanation for details). Please re-examine the importance of indicators based on the feedback results.
2. The importance scale rules still adopt the 1-9 scale method (same as the first round), and the filling requirements are consistent.

3. If you believe there is a significant difference between the preliminary weight ranking and your judgment, please adjust the scale values in the judgment matrix and explain the reasons in the feedback section.

III. Basic Information of Experts (You may use the information from the first round; please revise if there are any changes)

1. Research Field: □ Food Science □ Ideological and Political Education □ Education Evaluation □ Agricultural Policy □ Others ________
2. Professional Title: □ Professor □ Associate Professor □ Others ________
3. Work Experience: □ Less than 10 years □ 10-20 years □ More than 20 years
4. Familiarity with the Field of Big Food Concept: □ Very Familiar □ Relatively Familiar □ General □ Unfamiliar

IV. Indicator Explanation (Consistent with the First Round)

**Criterion Layer (Dimensions)**	**Indicator Layer (Specific Indicators)**	**Indicator Connotation**
Cognitive Dimension (B1)	Cognition of the Big Food Concept (C1)	Degree of understanding of the core definition and core characteristics of the Big Food Concept
	Cognition of the Diversified Food Supply System (C2)	Cognition of "full-field supply of agriculture, forestry, animal husbandry, and fishery", "forest grain depot", "blue granary", etc.
	Cognition of the Food Security Strategy (C3)	Understanding of national food security policies, self-sufficiency rate goals, etc.
	Cognition of Ecological Sustainability (C4)	Cognition of the coordinated relationship between food production and ecological protection
Affective Dimension (B2)	Emotion of Food Cherishing (C5)	Attitude of valuing food resources and emotional tendency to oppose waste
	Emotion of Gratitude for Labor (C6)	Respect and gratitude towards food producers
	Emotional Empathy for Ecology (C7)	Empathy and concern for food resource protection and ecological balance
	Emotional Identification with Policies (C8)	Emotional recognition and support for national policies related to the Big Food Concept

**Criterion Layer (Dimensions)**	**Indicator Layer (Specific Indicators)**	**Indicator Connotation**
Value Dimension (B3)	Healthy Diet Values (C9)	Tendency to take balanced nutrition and healthy consumption as the core values of diet
	Sense of Responsibility Values (C10)	Value judgment that regards ensuring food security as one's own social responsibility
	Values of Food Conservation and Environmental Protection (C11)	Value orientation advocating food conservation and reduction of resource consumption
	Values of Coordinated Development (C12)	Recognition of the value concept of "production-consumption-ecology" coordinated development
Behavioral Dimension (B4)	Food Conservation Behavior (C13)	Actual performance of reducing waste and ordering reasonably in daily diet
	Healthy Consumption Behavior (C14)	Consumption behavior of choosing nutritionally balanced, green and safe food
	Ecological Protection Behavior (C15)	Practical behaviors such as participating in garbage classification and supporting environmentally friendly food production
	Practical Participation Behavior (C16)	Behaviors of participating in research, labor practice, and publicity related to the Big Food Concept

V. Pairwise Comparison Judgment Matrix for Importance (Please Fill in Scale Values)

(I) Pairwise Comparison of the Criterion Layer (4 Dimensions)

**Criterion Layer Elements**	**Cognitive Dimension (B1)**	**Affective Dimension (B2)**	**Value Dimension (B3)**	**Behavioral Dimension (B4)**
Cognitive Dimension (B1)	-			
Affective Dimension (B2)	-	-		
Value Dimension (B3)	-	-	-	
Behavioral Dimension (B4)	-	-	-	-

(II) Pairwise Comparison of the Indicator Layer (Classified by Criterion Layer)

1. Comparison of Indicators under the Cognitive Dimension (B1)

Cognitive Dimension Indicators	Cognition of the Big Food Concept (C1)	Cognition of the Diversified Food Supply System (C2)	Cognition of the Food Security Strategy (C3)	Cognition of Ecological Sustainability (C4)
Cognition of the Big Food Concept (C1)	-			
Cognition of the Diversified Food Supply System (C2)	-	-		
Cognition of the Food Security Strategy (C3)	-	-	-	
Cognition of Ecological Sustainability (C4)	-	-	-	-

2. Comparison of Indicators under the Affective Dimension (B2)

Affective Dimension Indicators	Emotion of Food Cherishing (C5)	Emotion of Gratitude for Labor (C6)	Emotional Empathy for Ecology (C7)	Emotional Identification with Policies (C8)
Emotion of Food Cherishing (C5)	-			
Emotion of Gratitude for Labor (C6)	-	-		
Emotional Empathy for Ecology (C7)	-	-	-	
Emotional Identification with Policies (C8)	-	-	-	-

3. Comparison of Indicators under the Value Dimension (B3)

Value Dimension Indicators	Healthy Diet Values (C9)	Sense of Responsibility Values (C10)	Values of Food Conservation and Environmental Protection (C11)	Values of Coordinated Development (C12)
Healthy Diet Values (C9)	-			
Sense of Responsibility Values (C10)	-	-		
Values of Food Conservation and Environmental Protection (C11)	-	-	-	
Values of Coordinated Development (C12)	-	-	-	-

4. Comparison of Indicators under the Behavioral Dimension (B4)

Behavioral Dimension Indicators	Food Conservation Behavior (C13)	Healthy Consumption Behavior (C14)	Ecological Protection Behavior (C15)	Practical Participation Behavior (C16)
Food Conservation Behavior (C13)	-			
Healthy Consumption Behavior (C14)	-	-		
Ecological Protection Behavior (C15)	-	-	-	
Practical Participation Behavior (C16)	-	-	-	-

Thank you sincerely for your continuous support and professional guidance!

Research Group of "Construction of College Students' Big Food Concept Education Evaluation System and Research on Behavioral Impact Mechanism"

Questionnaire on College Students' Cognition and Behavior Regarding the Big Food Concept

Dear Students,

Greetings! The General Office of the State Council has issued the "Opinions on Practicing the Big Food Concept and Building a Diversified Food Supply System", elevating the Big Food Concept to a national strategic height. The Big Food Concept refers to a strategic concept for ensuring food security that breaks through the limitations of traditional staple foods, relies on the coordination of multiple industries including agriculture, forestry, animal husbandry, fishery, and grassland, integrates multiple dimensions of production, ecology, technology, and consumption, and achieves diversified and sustainable food supply.

This survey aims to understand the current status of college students' cognition, affect, values, and behaviors related to the Big Food Concept, and explore the practical impact of Big Food Concept education. The questionnaire is anonymous, and all data will only be used for academic research, with strict adherence to the principle of confidentiality. Please answer truthfully according to your actual situation. Thank you for your support and cooperation!

Filling Instructions: All items below adopt a 5-point Likert scale. For items in the Cognitive Dimension, the options are "1 = Completely Unaware, 2 = Slightly Unaware, 3 = Neutral, 4 = Relatively Aware, 5 = Very Aware"; for items in the Affective, Value, and Behavioral Dimensions, the options are "1 = Strongly Disagree, 2 = Slightly Disagree, 3 = Neutral, 4 = Relatively Agree, 5 = Strongly Agree". Please mark "√" on the number corresponding to your choice.

I. Basic Information

1. Gender: □1 = Male □2 = Female
2. Grade: □1 = Freshman □2 = Sophomore □3 = Junior □4 = Senior
3. Hometown: □1 = Inland Rural Area □2 = Inland Urban Area □3 = Coastal Urban Area
4. Family Monthly Total Income: □1 = 5,000 yuan and below □2 = 5,001 - 8,000 yuan □3 = 8,001 - 12,000 yuan □4 = 12,001 yuan and above
5. Name of Your University: ________

6. Have you received education related to the Big Food Concept (e.g., courses, lectures, publicity activities, etc.): □1 = Never □2 = Occasionally □3 = Frequently
7. The courses I participated in related to the Big Food Concept elaborated on the connotation of the diversified food supply system in detail: □1 = Never □2 = Occasionally □3 = Frequently
8. Practical activities (e.g., agricultural labor, visits to food supply chains) have effectively improved my understanding of the Big Food Concept: □1 = Never □2 = Occasionally □3 = Frequently
9. Campus publicity and education (posters, lectures, etc.) have enabled me to fully understand the core connotation of the Big Food Concept: □1 = Never □2 = Occasionally □3 = Frequently
10. Campus cultural edification (theme activities, community practices, etc.) has enhanced my willingness to practice the Big Food Concept: □1 = Never □2 = Occasionally □3 = Frequently

II. Core Items (42 Items in Total)

(I) Cognitive Dimension (12 Items)

1. I clearly understand the core definition and essence of the Big Food Concept □1 = Completely Unaware □2 = Slightly Unaware □3 = Neutral □4 = Relatively Aware □5 = Very Aware
2. I understand the historical background and policy orientation of the Big Food Concept □1 = Completely Unaware □2 = Slightly Unaware □3 = Neutral □4 = Relatively Aware □5 = Very Aware
3. I can accurately distinguish the differences between the Big Food Concept and traditional food concepts □1 = Completely Unaware □2 = Slightly Unaware □3 = Neutral □4 = Relatively Aware □5 = Very Aware
4. I clearly know the main components of China's diversified food supply system (e.g., grain, vegetables, meat, eggs, milk, marine food, etc.) □1 = Completely Unaware □2 = Slightly Unaware □3 = Neutral □4 = Relatively Aware □5 = Very Aware
5. I understand the key industries and technical support for ensuring diversified food supply □1 = Completely Unaware □2 = Slightly Unaware □3 = Neutral □4 = Relatively Aware □5 = Very Aware
6. I know the role of under-forest economy, protected agriculture, etc., in food supply □1 = Completely Unaware □2 = Slightly Unaware □3 = Neutral □4 = Relatively Aware □5 = Very Aware

7. I understand the core content and basic requirements of the national food security strategy □1 = Completely Unaware □2 = Slightly Unaware □3 = Neutral □4 = Relatively Aware □5 = Very Aware
8. I clearly understand the basic situation of China's grain supply and demand as well as the guarantee measures □1 = Completely Unaware □2 = Slightly Unaware □3 = Neutral □4 = Relatively Aware □5 = Very Aware
9. I know the impact of the international grain situation on China's food supply □1 = Completely Unaware □2 = Slightly Unaware □3 = Neutral □4 = Relatively Aware □5 = Very Aware
10. I understand the core requirements of ecological sustainable development in the Big Food Concept □1 = Completely Unaware □2 = Slightly Unaware □3 = Neutral □4 = Relatively Aware □5 = Very Aware
11. I clearly understand the balanced relationship between agricultural production and ecological environmental protection □1 = Completely Unaware □2 = Slightly Unaware □3 = Neutral □4 = Relatively Aware □5 = Very Aware
12. I know the long-term impact of over-development on the sustainability of food supply □1 = Completely Unaware □2 = Slightly Unaware □3 = Neutral □4 = Relatively Aware □5 = Very Aware

(II) Affective Dimension (10 Items)

13. When I see food waste, I feel regretful and distressed □1 = Strongly Disagree □2 = Slightly Disagree □3 = Neutral □4 = Relatively Agree □5 = Strongly Agree
14. When eating food, I feel grateful to the producers □1 = Strongly Disagree □2 = Slightly Disagree □3 = Neutral □4 = Relatively Agree □5 = Strongly Agree
15. After learning about the hardships of food production, I will cherish food more □1 = Strongly Disagree □2 = Slightly Disagree □3 = Neutral □4 = Relatively Agree □5 = Strongly Agree
16. I have great respect for farmers and workers in the food production and circulation links □1 = Strongly Disagree □2 = Slightly Disagree □3 = Neutral □4 = Relatively Agree □5 = Strongly Agree
17. I feel worried when I learn that farmland ecological damage affects food supply □1 = Strongly Disagree □2 = Slightly Disagree □3 = Neutral □4 = Relatively Agree □5 = Strongly Agree
18. I feel a sense of identification with the policy measures to ensure food security □1 = Strongly Disagree □2 = Slightly Disagree □3 = Neutral □4 = Relatively Agree □5 = Strongly Agree

19. I gain psychological satisfaction when participating in food conservation activities □1 = Strongly Disagree □2 = Slightly Disagree □3 = Neutral □4 = Relatively Agree □5 = Strongly Agree
20. After understanding the connotation of the Big Food Concept, I have an emotional resonance □1 = Strongly Disagree □2 = Slightly Disagree □3 = Neutral □4 = Relatively Agree □5 = Strongly Agree
21. I feel happy when I see the good development of green ecological agriculture □1 = Strongly Disagree □2 = Slightly Disagree □3 = Neutral □4 = Relatively Agree □5 = Strongly Agree
22. I feel disgusted with behaviors that damage the ecological environment of food supply □1 = Strongly Disagree □2 = Slightly Disagree □3 = Neutral □4 = Relatively Agree □5 = Strongly Agree

(III) Value Dimension (10 Items)

23. I believe that healthy eating is an important manifestation of practicing the Big Food Concept □1 = Strongly Disagree □2 = Slightly Disagree □3 = Neutral □4 = Relatively Agree □5 = Strongly Agree
24. I adhere to the value concept of "saving food and opposing waste" □1 = Strongly Disagree □2 = Slightly Disagree □3 = Neutral □4 = Relatively Agree □5 = Strongly Agree
25. I believe that ensuring food security is the responsibility and obligation of every citizen □1 = Strongly Disagree □2 = Slightly Disagree □3 = Neutral □4 = Relatively Agree □5 = Strongly Agree
26. I agree with the food production value orientation of "harmonious coexistence between humans and nature" □1 = Strongly Disagree □2 = Slightly Disagree □3 = Neutral □4 = Relatively Agree □5 = Strongly Agree
27. I believe that low-carbon environmental protection should be integrated into daily dietary consumption □1 = Strongly Disagree □2 = Slightly Disagree □3 = Neutral □4 = Relatively Agree □5 = Strongly Agree
28. I adhere to the value principle of "balanced diet and moderate nutrition" □1 = Strongly Disagree □2 = Slightly Disagree □3 = Neutral □4 = Relatively Agree □5 = Strongly Agree
29. I believe that individual behaviors should serve the overall situation of national food security □1 = Strongly Disagree □2 = Slightly Disagree □3 = Neutral □4 = Relatively Agree □5 = Strongly Agree
30. I agree with the values of coordinated development of food production, economy, and ecology □1 = Strongly Disagree □2 = Slightly Disagree □3 = Neutral □4 = Relatively Agree □5 = Strongly Agree

31. I believe that we should take the initiative to spread the core value concept of the Big Food Concept □1 = Strongly Disagree □2 = Slightly Disagree □3 = Neutral □4 = Relatively Agree □5 = Strongly Agree
32. I adhere to the dietary value criterion of "cherishing resources and moderate consumption" □1 = Strongly Disagree □2 = Slightly Disagree □3 = Neutral □4 = Relatively Agree □5 = Strongly Agree

(IV) Behavioral Dimension (10 Items)

33. I practice "clean plate" when dining to avoid food waste □1 = Strongly Disagree □2 = Slightly Disagree □3 = Neutral □4 = Relatively Agree □5 = Strongly Agree
34. I take the initiative to choose green and organic food products □1 = Strongly Disagree □2 = Slightly Disagree □3 = Neutral □4 = Relatively Agree □5 = Strongly Agree
35. I remind people around me to save food and oppose wasteful behaviors □1 = Strongly Disagree □2 = Slightly Disagree □3 = Neutral □4 = Relatively Agree □5 = Strongly Agree
36. I take the initiative to learn about policies and knowledge related to the Big Food Concept □1 = Strongly Disagree □2 = Slightly Disagree □3 = Neutral □4 = Relatively Agree □5 = Strongly Agree
37. I participate in garbage classification to reduce food-related environmental pollution □1 = Strongly Disagree □2 = Slightly Disagree □3 = Neutral □4 = Relatively Agree □5 = Strongly Agree
38. I reasonably purchase food according to my own needs to avoid excessive hoarding □1 = Strongly Disagree □2 = Slightly Disagree □3 = Neutral □4 = Relatively Agree □5 = Strongly Agree
39. I take the initiative to participate in publicity or practical activities related to the Big Food Concept □1 = Strongly Disagree □2 = Slightly Disagree □3 = Neutral □4 = Relatively Agree □5 = Strongly Agree
40. I refuse to eat wild animals and illegal food products □1 = Strongly Disagree □2 = Slightly Disagree □3 = Neutral □4 = Relatively Agree □5 = Strongly Agree
41. I prioritize local and seasonal food to reduce transportation energy consumption □1 = Strongly Disagree □2 = Slightly Disagree □3 = Neutral □4 = Relatively Agree □5 = Strongly Agree
42. I take the initiative to explain the core content of the Big Food Concept to others □1 = Strongly Disagree □2 = Slightly Disagree □3 = Neutral □4 = Relatively Agree □5 = Strongly Agree

III. Closing Remarks

Congratulations on completing all items! Thank you again for taking the precious time to participate in this survey. Wish you academic progress and a happy life!
